# Glutamine metabolic reprogramming in acute and chronic kidney diseases: mechanistic insights and therapeutic implications

**DOI:** 10.3389/fimmu.2026.1939528

**Published:** 2026-10-12

**Authors:** Ruotong Xu, Jin Zhao, Minna Liu, Yifan Qiao, Xiaoxuan Ning, Shiren Sun

**Affiliations:** 1 Department of Nephrology, Xijing Hospital, Fourth Military Medical University, Xi’an, China; 2 Shaanxi Provincial Clinical Research Center for Kidney Diseases, Xi’an, China; 3 Fundamental Medical Science Research Laboratories, the 940th Hospital Joint Logistics Support Forces of the People’s Liberation Army, Lanzhou, China; 4 Department of Geriatric, Xijing Hospital, Fourth Military Medical University, Xi’an, China

**Keywords:** acute kidney injury, chronic kidney disease, fibrosis, glutaminase 1, glutamine metabolic reprogramming, immunometabolism, inflammation

## Abstract

Acute kidney injury (AKI) and chronic kidney disease (CKD) are common clinical disorders that have high incidence and mortality. Their pathogenesis and the mechanisms underlying AKI-to-CKD progression remain unclear. Metabolic reprogramming has emerged as a core mechanism driving the onset and progression of kidney diseases. Glutamine (Gln) metabolism, a central component of renal energy metabolism, not only maintains acid-base balance and cellular homeostasis but also actively participates in pathophysiological processes including ischemic injury, oxidative stress, inflammatory regulation and fibrosis. Multi-omics studies have revealed stage-specific alterations in Gln metabolism-associated signatures during kidney injury. In AKI, altered Gln levels and redox-related metabolites may suggest disturbances in metabolic pathways, whereas in CKD, elevated glutaminase 1 (GLS1) expression and glutaminolysis-associated metabolic reprogramming are associated with fibrosis. Gln, as an immunonutrient, also serves as a critical energy substrate to sustain the proliferation, activation, and effector functions of immune cells. Gln metabolism may reshape the immune microenvironment within the injured kidney, thereby modulating disease progression. Targeting the Gln metabolic axis may offer new therapeutic insights for AKI and CKD. However, the changes in Gln metabolism and its consequent immunometabolic effects during the AKI-to-CKD transition remain poorly understood. This narrative review aims to provide a comprehensive overview of the dynamic changes in Gln metabolism during acute and chronic kidney diseases, its stage-specific mechanisms, and its potential as a therapeutic target. We also seek to provide a theoretical basis for developing novel biomarkers and targeted therapies based on Gln metabolic reprogramming.

## 1 Introduction

Chronic kidney disease (CKD), a major global public health challenge, is receiving increasing focus driven by the dual trends of population aging and the rising worldwide prevalence of hypertension and diabetes (1). In 2023, an estimated 788 million adults worldwide were living with CKD, corresponding to a global age-standardized prevalence of 14.2% (2). In China, its prevalence has risen to around 10% (3). Some patients are prone to progressing to end-stage renal disease (ESRD), which is closely associated with a high disease burden and mortality. CKD causes approximately 1.2 million deaths annually, and is estimated to rise to the fifth leading cause of death globally by 2040 (4). CKD has complex etiologies and insidious onset, making early diagnosis challenging and leaving a lack of effective prevention or intervention targets (5). Of note, acute kidney injury (AKI) is a major risk factor for the development of CKD and even end-stage renal disease ESRD. Experiencing an AKI significantly elevates the risk of subsequently developing CKD and progressing to ESRD (6). Even mild or transient AKI may increase the risk of subsequent CKD and long-term adverse outcomes. AKI is generally associated with increased mortality (7, 8).

The etiology of kidney injury is multifaceted and heterogeneous (9). As a vital organ for energy metabolism, the kidney often exhibits significant metabolic dysfunction following injury. Recently, metabolomics has contributed significantly to research on kidney diseases. Research indicates that metabolic disturbances are not only crucial pathological features of acute and chronic kidney diseases and the progression from AKI to CKD, but also key contributors to their pathogenesis and progression (10–12). Among various metabolic disorders, amino acid metabolic dysregulation may represent a core gap. On the one hand, as fundamental units of protein metabolism, the homeostasis imbalance of amino acids directly reflects the extent of renal metabolic dysfunction (13). On the other hand, metabolic abnormalities of specific amino acids may exacerbate renal tubular epithelial cells (TECs) injury through multiple pathways, including impacts on energy metabolism, oxidative stress, and inflammatory responses (13, 14). This mechanism establishes amino acid metabolic dysregulation not only as a significant metabolic characteristic, but also provides potential biomarkers for early diagnosis and opens new avenues for developing targeted therapeutic strategies based on amino acid metabolism regulation. Research on specific amino acid metabolic disorders has been a hot topic in recent years. Glutamine (Gln) is the most abundant free amino acid in plasma. It not only meets basic nutritional needs but also serves as an important energy source for immune cells. Meanwhile, Gln participates in the regulation of inflammatory responses and oxidative stress. Interestingly, recent studies have revealed that the Gln metabolic axis may be closely associated with AKI and CKD (15).

In the early stages of AKI, Gln metabolism undergoes significant remodeling, characterized by decreased Gln levels in the circulation and renal tissue, suggesting enhanced Gln catabolism (16–18). This depletion not only reflects a state of early oxidative stress and a cellular metabolic crisis, but also exacerbates these pathological changes. We speculate that, in the context of specific AKI, targeting this stage via exogenous supplementation of Gln or its metabolites appears to be a rational therapeutic strategy. Recently, this approach has once again shown promise in mitigating kidney injury in some preclinical models and exploratory clinical studies. Although controversy exists regarding earlier studies in critically ill patients, these findings have refocused our attention on Gln (14, 19). During the CKD and fibrotic phase, upregulation of the key enzyme glutaminase 1 (GLS1) drives glutaminolysis to meet the heightened biosynthetic demands of activated cells such as fibroblasts (20). This suggests that, as opposed to exogenous Gln supplementation, in chronic fibrotic settings, certain cell subpopulations may exhibit enhanced Gln catabolic features, and selective modulation of the relevant metabolic enzymes could theoretically delay fibrosis progression by limiting the metabolic adaptation and biosynthetic demands of pro-fibrotic cells (20).

Two recently published studies also suggest this stage-specific role of Gln. In the context of ischemia-reperfusion injury (IRI), spermine acts as an endogenous iron chelator (21). It inhibits lipid peroxidation and ferroptosis by chelating Fe^2+^, thereby attenuating IRI damage in organs such as the kidney. However, spermine synthesis depends on Gln as a nitrogen source via the non-canonical pathway mediated by aldehyde dehydrogenase 18 family member A1 (ALDH18A1). This finding suggests a potential contribution of Gln metabolism to renal protection. On the other hand, hyperglutaminolysis serves as a driver of aging. Upregulated GLS1 activity enhances Gln breakdown (22). The resulting glutamate (Glu) and ammonium ions promote arginine synthesis. This, in turn, relieves the inhibition of mammalian target of rapamycin complex 1 (mTORC1) by cytosolic arginine sensor for mTORC1 subunit 1 (CASTOR1). This leads to sustained mTORC1 activation and ultimately accelerates cellular senescence and organ aging. Aging is a critical promoter of the AKI-to-CKD transition. Hyperglutaminolysis may thus act as a key metabolic switch in this malignant transformation. It potentially induces senescence in TECs, promotes metabolic reprogramming, and sustains a pro-inflammatory phenotype. Therefore, targeting the GLS1-arginine-mTORC1 axis or modulating the spermine-mediated ferroptosis pathway may provide a promising novel strategy to delay kidney disease progression. Immune cells are critical regulators of the onset and progression of AKI (23), and the potential role of immunometabolism in AKI pathogenesis has gained increasing attention. Gln metabolism is closely associated with immune cell proliferation, activation, and functional regulation (24, 25). As a highly metabolically demanding process, fibrosis is also dynamically regulated by immune cells (23). Therefore, elucidating the dynamic regulatory mechanisms of Gln immunometabolism during renal injury, repair, and fibrosis may provide new theoretical insights and research directions for the development of therapeutic strategies targeting immunometabolic pathways. Previous studies have provided a broad conceptual framework for Gln catabolism as a therapeutic target in kidney diseases (26). However, they have not fully explored how the Gln metabolic pathway serves as a bridge linking metabolism and immunity in acute and chronic kidney diseases. Specifically, it remains unclear how Gln metabolism mediates disease processes by regulating metabolic reprogramming of renal TECs, immune cell proliferation and differentiation, inflammatory responses and fibrotic processes. In this narrative review, we comprehensively discuss Gln metabolism in acute and chronic kidney diseases from mechanistic, clinical, and translational perspectives. By examining the dynamic metabolic alterations of Gln during AKI and CKD, we further explore the mechanisms through which this metabolite contributes to disease progression and its interplay with immune-inflammatory processes. This review aims to provide new insights into the mechanisms underlying AKI-to-CKD progression, clarify currently unresolved issues, and highlight potential directions for future research.

## 2 Overview of Gln

### 2.1 Gln metabolic pathway

Gln is defined as a non-essential amino acid that exists at the highest concentration among free amino acids in the blood. It participates in purine, pyrimidine, and nucleic acid synthesis and the tricarboxylic acid (TCA) cycle and also produces glutathione (GSH) (15). However, under stress or pathological conditions, endogenous Gln synthesis may not meet the body’s demands, requiring exogenous supplementation. Therefore, it is also known as a conditionally essential amino acid (15, 27). The Gln metabolism pathway has been characterized as playing a central role in inflammation, redox homeostasis, immune regulation, maladaptive repair, cell survival, and proliferation (26).

As illustrated in **Figure 1**, Gln enters renal TECs via alanine-serine-cysteine transporter 2 (ASCT2) or solute carrier family1 member 5 (SLC1A5) on the cytosol membrane. The SLC1A5 variant, which serves as the mitochondrial Gln transporter, mediates the transport of Gln across the inner mitochondrial membrane. Subsequently, Gln is transformed into Glu (28). Part of the Glu synthesized via catabolism can enter the cytoplasm via transporters, and part remains in the mitochondria for further metabolism. Cytoplasmic Glu is involved in the biosynthesis of the antioxidant GSH and non-essential amino acids such as proline and aspartate. It acts as an exchange factor for input of extracellular cystine via solute carrier family 7 member 11(SLC7A11). Mitochondrial Glu then subsequently achieves conversion to α-ketoglutarate(α-KG) via Glu dehydrogenase (GLUD) or a group of aminotransferases, including Glutamate-pyruvate transaminase (GPT), Glutamate-oxaloacetate transaminase (GOT), and phosphoserine aminotransferase (PSAT) (29).

**Figure 1:**
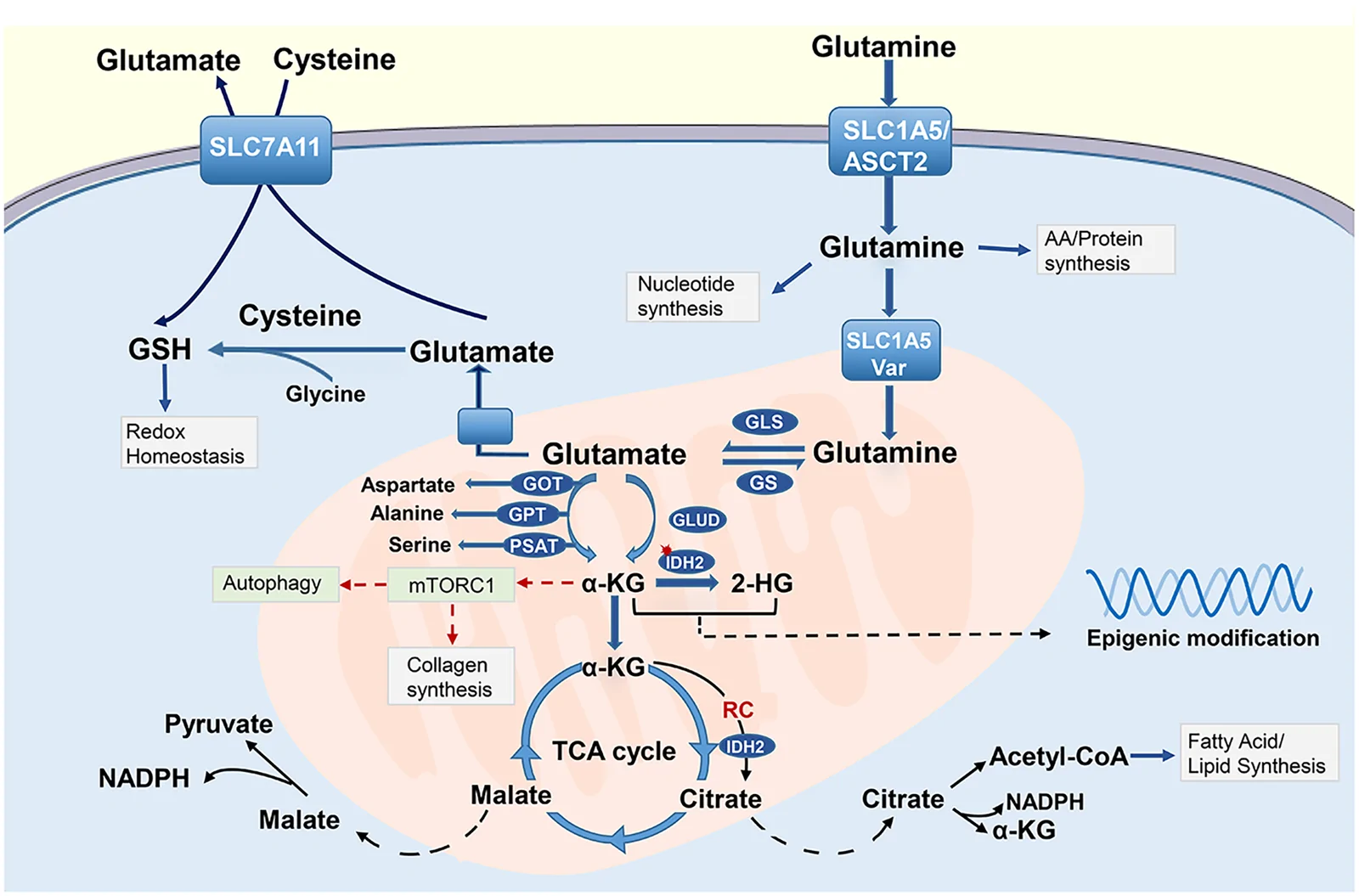
Gln metabolites and the interlinked signaling pathways. Gln enters cells via the SLC1A5/ASCT2 transporter on the cell membrane and participates in the synthesis of essential intracellular substances, such as proteins and nucleotides. Within the mitochondria, Gln is converted to Glu by GLS and further metabolized to produce α- KG. α- KG not only enters the TCA cycle to generate energy but also, along with 2-HG, regulates epigenetic modifications through α-KG-dependent dioxygenases. Additionally, GSH, synthesized from cytoplasmic Glu, plays a critical role in maintaining intracellular redox homeostasis. SLC1A5/ASCT2, Solute Carrier Family 1 Member 5/Alanine-Serine-Cysteine Transporter 2; AA, Amino Acid; SLC1A5Var, Solute Carrier Family 1 Member 5 Variant; GLS/GLS2, glutaminase/glutaminase2; GLUD, Glutamate dehydrogenase; GOT, Glutamte-oxaloacetate transaminase; GPT, Glutamate–pyruvate transaminase; PSAT, phosphoserine transaminase; IDH2, isocitrate dehydrogenase 2; α-KG, α-ketoglutarate; 2-HG, 2-hydroxyglutaric acid; TCA cycle, tricarboxylic acid cycle; RC, reductive carboxylation; GSH, glutathione; SLC7A11, Solute Carrier Family 7 Member 11.

α-KG serves as a pivotal metabolite involved in ATP production and the replenishment of TCA cycle intermediates via anaplerosis. Additionally, α-KG promotes the synthesis of 2-hydroxyglutarate (2-HG). Both α-KG and 2-HG can modulate epigenetic modifications through the action of α-KG-dependent dioxygenases (30). Under conditions of hypoxia or mitochondrial dysfunction, isocitrate dehydrogenase 2 (IDH2) catalyzes a reductive carboxylation reaction that converts α - KG into citrate. When the environment is hypoxic or when hypoxia - inducible factor (HIF)- 2α is in a stabilized state, the newly - formed citrate leaves the mitochondria and is then employed for the synthesis of fatty acids, amino acids, and the reducing agent reduced nicotinamide adenine dinucleotide phosphate (NADPH) (28). In addition, α-KG activates the mTORC1 after being translocated into the cytoplasm, which promotes collagen synthesis by activating prolyl-4-hydroxylase(P4H) (31).

### 2.2 The role of Gln in kidney

Gln serves as one of the primary gluconeogenic amino acids (32, 33). It contributes approximately 20% to 25% of systemic glucose production, enhancing net muscle glycogen storage and stimulating gluconeogenesis in healthy individuals (34). α-KG, a key intermediate in the TCA cycle and oxidative phosphorylation, supports lipid and NADH synthesis (19, 28) and provides substrates for lactate production, which in turn maintains fatty acid oxidation (FAO) by fine-tuning the NAD+/NADH ratio (35). Additionally, α-KG regulates cellular histone and DNA methylation levels (36). As a nitrogen source, Gln directly participates in the biosynthesis of nucleotides, glucosamine, and other non-essential amino acids. Its γ-nitrogen atom is critical in five reactions of *de novo* nucleotide synthesis (37) and indirectly contributes to synthesis by providing amino groups or metabolic intermediates, such as generating Glu with amino groups from aspartate (38). GSH derived from Gln is primarily involved in the regulation of redox homeostasis. Gln itself is also involved in interorgan nitrogen transport, ammonia detoxification, and the regulation of immune and inflammatory responses (39).

Furthermore, as an organ with high metabolic demands, the kidney relies on tightly regulated energy metabolic processes to maintain function, and Gln metabolism plays a crucial role in this process (40, 41).

Gln metabolism plays a pivotal role in sustaining systemic homeostasis. Gln is primarily reabsorbed in the proximal tubule (PT) of the kidney, where it serves as the major metabolic substrate for renal ammoniagenesis. Each molecule of Gln catabolized generates equimolar amounts of ammonium (NH_4_^+^) and bicarbonate (HCO_3_^-^) ions. The bicarbonate produced is subsequently transported into the systemic circulation, where it plays a central role in neutralizing the daily endogenous acid load, thereby constituting a core mechanism for the maintenance of systemic acid-base homeostasis (42). Therefore, tightly regulated renal Gln metabolism and the subsequent directional transport of ammonia collectively determine urinary ammonium excretion. This process constitutes the final regulatory step for net acid excretion and systemic acid-base balance, ultimately contributing to cellular and whole-body homeostasis (42). This process is initiated by the highly expressed, phosphate-dependent glutaminase (PDG). Mammals possess two distinct *GLS* genes: *GLS1* and *GLS2*( *(*43). While *GLS2* is predominantly and highly expressed in the liver, *GLS1* exhibits a broader tissue distribution. The *GLS1* gene encodes two principal isoforms through alternative splicing: kidney-type glutaminase (KGA), which is specifically expressed in the kidney and mediates ammonia production for acid excretion, and the glutaminase C (GAC) isoform. GAC is markedly upregulated in numerous cancer cell types and is strongly implicated in supporting the glutaminolytic metabolism of tumor cells (44). As a tractable therapeutic target, *GLS1* has led to the development of various inhibitors for cancer treatment (45).

## 3 Gln metabolism in AKI

AKI can cause significant changes in Gln metabolism (12, 17). At the level of circulating metabolites, experimental IRI models have shown that IRI leads to a significant decrease in plasma Gln availability, suggesting that Gln metabolism is involved in IRI-associated metabolic reprogramming (17). Metabolomic analyses conducted in clinical patients have also found that, among patients treated with cisplatin, those who developed AKI had lower serum Gln levels than those who did not develop AKI (46). At the tissue metabolite level, Gln was significantly downregulated in the kidney tissue of the rat IRI model at both 24 and 72 hours after reperfusion (16). A persistent decrease in Gln levels in the renal cortex was also observed in an IRI-induced AKI mouse model (17). The LPS-induced sepsis-associated acute kidney injury (SA-AKI) model also exhibited significant renal metabolic reprogramming, suggesting that Gln metabolism may be commonly regulated during AKI (47). The above preclinical and clinical evidence suggests that Gln levels tend to decrease both systemically and locally in the kidneys. At the gene expression level, Nowik et al. observed an upregulation of the Gln transporter sodium-coupled neutral amino acid transporter 3 (SNAT3) and enzymes involved in Gln catabolism and ammonia production in the renal proximal tubules of an NH_4_ Cl-induced mouse model of metabolic acidosis (MA). This was accompanied by increased urinary ammonium excretion, suggesting enhanced Gln uptake and catabolism in the kidney. These adaptive changes help maintain acid-base balance (48). Single-cell transcriptomics studies have revealed that the expression of the Gln catabolism-related genes Gls and Glud1 is upregulated in the PT of unilateral ischemia-reperfusion injury (uIRI) and unilateral ureteral obstruction (UUO) mice (49, 50). However, due to limitations in the clinical setting, genetic-level changes have not yet been supported in clinical studies. Based on existing preclinical and clinical evidence, we can infer that decreased levels of Gln metabolites in the circulation and tissues, upregulation of catabolic enzyme gene expression, and increased tissue uptake indicate that Gln metabolism is reprogrammed in AKI. These alterations reflect changes in the circulatory system, tissue parenchymal cell metabolism, gene expression regulation, suggesting that Gln metabolism may serve as a key metabolic node in AKI pathogenesis.

Gln functions as both a metabolic substrate and an immunomodulatory molecule and may exert diverse effects in response to AKI. **Figure 2** integrates established mechanisms of Gln metabolism with protective effects observed following exogenous Gln supplementation in AKI models.

**Figure 2:**
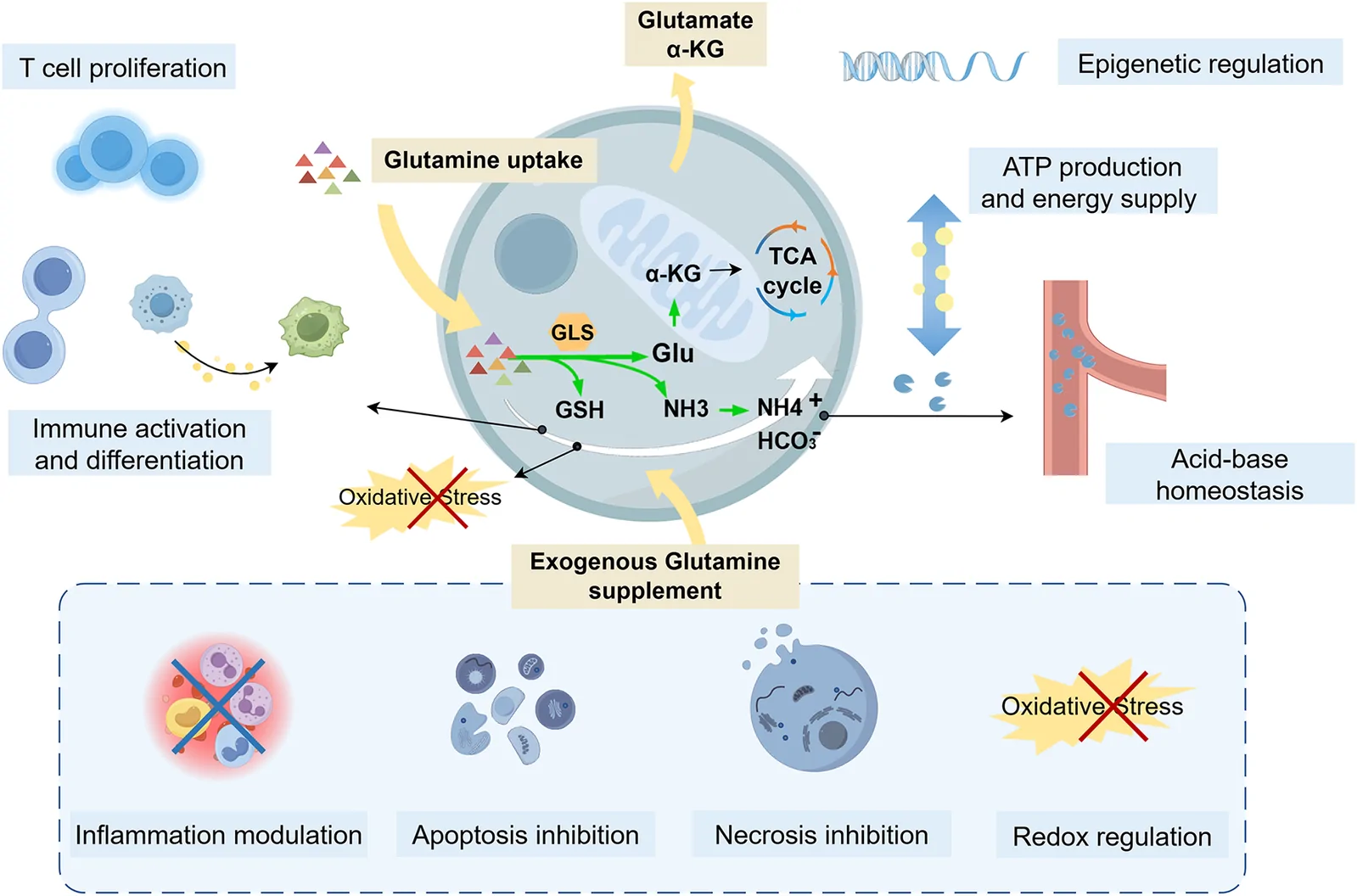
Potential role of Gln metabolism in tubular responses after acute kidney injury. Gln metabolism provides substrates for energy production through glutaminolysis and the TCA cycle, contributes to redox homeostasis through GSH synthesis, and participates in renal acid–base regulation through ammoniagenesis. In addition, Gln serves as an important regulator of immune cell metabolism, influencing immune cell proliferation, activation, and differentiation in a context-dependent manner. Experimental studies using exogenous Gln supplementation in AKI models have demonstrated protective effects, including attenuation of oxidative stress, tubular cell death, and inflammatory responses. GLS, glutaminase; Glu, Glutamte; GSH, glutathione; α-KG, α-ketoglutarate; TCA cycle, tricarboxylic acid cycle.

### 3.1 Gln and renal TECs

Tissue damage in AKI primarily occurs in renal TECs, especially in the metabolically active PT (51, 52). TECs undergo a profound metabolic crisis during AKI. Direct insults such as ischemia and toxins induce mitochondrial dysfunction, which disrupts the TCA cycle and impairs ATP production. This resultant energy deficit and the need for tissue repair together lead to metabolic reprogramming (53, 54). Consequently, Gln, as the most abundant amino acid in circulation, may therefore be recruited and consumed in a compensatory manner. Experimental studies using cellular and animal models have shown that Gln demand increases and Gln availability may decrease under AKI stress conditions, potentially affecting the injury response of TECs. Study conducted using a mouse model of IRI-induced AKI has shown that exogenous Gln supplementation significantly improves renal function, reduces injury biomarkers, and alleviates histopathological damage. In clinical settings, exogenous Gln supplementation significantly reduced biomarkers of kidney injury in high-risk AKI patients following cardiac surgery (14, 19). These findings suggest that changes in Gln availability may be an important metabolic feature of AKI and support the potential protective role of Gln supplementation in TECs injury.

The protective effects of Gln are mediated through several mechanisms, the first of which is its role as a metabolic substrate for TECs. In IRI-induced AKI, the increased expression of transforming growth factor (TGF)-β1 in renal TECs leads to the upregulation of GLS1, promoting Gln catabolism and the generation of α-KG. The produced α-KG, serving as a supplement to the TCA cycle, can meet the elevated cellular energy demands required to maintain ionic balance (55). Gln also increases ATP concentration and the NAD^+^/NADH ratio to improve mitochondrial function and reduce the accumulation of mitochondrial reactive oxygen species (ROS) in TECs, thereby alleviating oxidative stress (14). The study found that in rats with glycerol-induced rhabdomyolytic AKI, Gln treatment also reduced oxidative stress by significantly increasing GSH levels in kidney tissue (56).

Gln can also attenuate TEC apoptosis in the early stages of AKI and reduce TEC loss. Specifically, in rats with glycerol-induced rhabdomyolytic AKI, Gln may reduce TEC apoptosis, particularly in the distal tubules, by upregulating the expression of heat shock protein 70 (Hsp70), inhibiting the c-Jun N-terminal kinase (JNK) signaling pathway, and modulating interactions among mitochondrial-associated proteins (56). This effect is also achieved by significantly reducing the expression of apoptosis-related proteins, such as programmed cell death protein (Pdcd) 6 and Pdcd10, in renal tissue (14). Additionally, it modulates the phosphorylation status of key proteins, including promoting phosphorylation at the Ser136 site of B-cell lymphoma 2 (BCL2)-associated agonist of cell death (Bad), reducing the phosphorylation levels of JNK and 14-3-3ζ, and inhibiting caspase-3 activation, thereby inhibiting the mitochondrial-mediated intrinsic apoptotic pathway (14). LI et al. proposed that the protective effect of Gln against I/R-induced AKI may be achieved through the regulation of microRNAs (miRNAs), with the cyclic guanosine monophosphate-protein kinase G (cGMP-PKG) signaling pathway mediated by microRNA-132-5p(miR-132-5p) serving as a potential mechanism. In this experimental study, miR-132-5p expression was downregulated in the I/R group but was restored following Gln intervention, thereby exerting an anti-apoptotic effect via the cGMP-PKG signaling pathway and achieving renal protection (57). Furthermore, using a human proximal renal tubular epithelial cell (HK-2) hypoxia/reoxygenation (A/R) model, it was found that this anti-apoptotic effect of Gln may also be mediated by inducing heme oxygenase-1 (HO-1) expression via a p38 mitogen-activated protein kinase (MAPK)-dependent pathway (58). In cisplatin-induced AKI rats, Gln administration was also observed to reduce renal tubular necrosis and tubular pattern formation, and it significantly reduced the number of terminal deoxynucleotidyl transferase dUTP nick-end labelling(TUNEL)-positive cells and cleaved caspase-3 expression in the TECs, suggesting that it does have anti-apoptotic activity (59). Lu et al. also reported that TECs undergo phenotypic switching during repair and proliferation, and considered that Gln may play a role in this process (60).

### 3.2 Gln and immune cells

Complex inflammatory processes within the kidney, driven by lymphocytes and innate immune cells, play a key role in the onset and progression of AKI (23). While some immune cells, such as CD4-positive T lymphocytes (CD4^+^T cells), contribute to disease progression, others, such as Regulatory T cells (Tregs), play a protective role. Meanwhile, macrophages and Natural killer (NK) cells exhibit varying roles depending on the course of the disease (23). The complex interactions between immune cells and renal cells influence the outcome of kidney injury (61). Metabolism is a central mechanism underlying the activation and differentiation of immune cells (62). Among metabolic pathways, Gln metabolism, similar to glycolysis and fatty acid oxidation, is not merely a downstream consequence of cellular function but also serves as a metabolic checkpoint and regulator of immune cell function (63). Several studies on Gln metabolism conducted in the 1980s by Eric Newsholme’s team confirmed that Gln has a consumption rate similar to or higher than glucose in immune cells, Gln is considered the “fuel of the immune system” (15, 64). Currently, therapeutic strategies targeting immune metabolism are a rapidly evolving field. Gln metabolism in immune cells may represent a potential therapeutic target in the onset and progression of AKI.

#### 3.2.1 Gln metabolism in T cells

Studies have shown that T cells can be recruited to the kidney early after ischemia and persist during the repair phase following injury, playing a role in regulating the renal inflammatory response and repair process (65, 66). Metabolic reprogramming is a key mechanism underlying T-cell activation and differentiation (23). Existing studies in immunometabolism has shown that activated T cells utilize aerobic Gln metabolism to meet the biosynthetic and energy demands of rapid proliferation (67–69). Gln catabolism helps T lymphocytes maintain redox homeostasis by promoting GSH synthesis, thereby supporting the differentiation and survival of effector T cells (67, 70). Inhibition of GLS-1 reduces T-cell proliferation and the expression of activation-related markers (31). Therefore, the Gln metabolic pathway is considered one of the key metabolic checkpoints regulating T-cell function. However, the specific role of T-cell Gln dependence in kidney diseases remains to be further validated using disease models.

Lee et al. (71) found that renal T cells in AKI undergo significant metabolic reprogramming using an IRI mouse model. GLS1 activity in renal T cells was increased during the ischemic phase compared with controls and returned to baseline after reperfusion, showing time-dependent changes. Inhibition of GLS1 using JHU083 significantly reduced creatinine and Neutrophil Gelatinase-Associated Lipocalin (NGAL) levels and also attenuated functional and structural kidney injury in the cisplatin model. JHU083 drove renal CD4^+^ and CD8^+^ T cells toward an immature phenotype, reduced Proliferation marker protein antigen identified by monoclonal antibody Ki-67 (Ki-67) expression, and dose-dependently suppressed CD3/CD28-mediated T-cell activation and proliferation *in vitro*. This protective effect was dependent on T cells, as Gln blockade failed to provide protection in T-cell-deficient forkhead box N1; nude (Foxn1^nu^) mice. It is worth noting that protective double-negative (DN) T cells were relatively tolerant to Gln blockade, and their activation and proliferation were even slightly enhanced (71). Further research by the team found that JHU083 enhanced the protective effects of DNT cells, reduced effector CD4^+^ T-cell activation, and decreased renal fibrosis by restoring T-cell metabolism (72). These subpopulation-specific differences suggest that the effects of Gln metabolic regulation on the AKI immune response are cell-type-specific. Therefore, metabolic intervention strategies targeting different T-cell subsets require further refinement and validation. However, in mice with gut-derived polymicrobial sepsis, Gln supplementation maintained the proportions of T cells and CD4^+^ T cells in the circulation and spleen, reduced interleukin-4^+^ (IL-4^+^) and Forkhead box protein 3 (Foxp3^+^) T-cell subsets, and corrected immune dysregulation. It also upregulated the expression of the anti-apoptotic protein Bcl-2 while downregulating renal Kidney Injury Molecule-1 (Kim-1), Keratinocyte-derived Chemokine (KC), and Keratinocyte-derived Chemokine (MIP-2) expression, thereby alleviating remote kidney injury (73). In summary, the effects of Gln on T cells are context-dependent. In AKI characterized by excessive activation of effector T cells, enhanced Gln metabolism supports effector T-cell function, therefore, targeting this metabolic axis may represent a potential therapeutic strategy. In contrast, in sepsis accompanied by lymphocyte depletion, Gln may help maintain T-cell subset balance by supporting T-cell survival and metabolic adaptation. Therefore, interventions targeting the same metabolic axis should be tailored according to immune status and disease stage.

#### 3.2.2 Gln metabolism in macrophages

Animal models suggest that macrophages are major contributors to the inflammatory response in AKI (74). In a mouse IRI model, renal macrophage numbers increased within 24 hours after reperfusion. Clearance experiments conducted at different time points indicated that M1 pro-inflammatory macrophages primarily promoted tubular injury during the early stage, whereas M2 macrophages played a key role in tubular proliferation and cellular repair during the later stage. Sustained M2 macrophage activation contributed to fibrosis (74). Previous studies suggest that this phenotypic transformation is closely associated with metabolic alterations, with Gln and arginine (Arg) metabolism playing a key role in these functional and phenotypic changes (75).

Currently, evidence regarding the mechanisms by which Gln regulates macrophage polarization is derived primarily from *in vitro* studies. In bone marrow-derived macrophages polarized with IL-4 or lipopolysaccharide (LPS), U-^13^C-Gln tracing revealed that approximately one-third of the carbon in TCA cycle metabolites in anti-inflammatory macrophages was derived from Gln (75, 76). IL-4-specific activation of M2 macrophages is essential for promoting TEC proliferation, and Gln appears to be a key factor in this activation process. α-KG, generated from Gln catabolism, promotes M2 gene expression through FAO and Jmjd3-dependent histone demethylation. This process establishes a metabolic checkpoint whereby Gln regulates M2 activation, a high α-KG/succinate ratio favors M2 polarization, whereas a low ratio promotes M1 polarization (77). Moreover, Gln was found to modulate the synthesis and secretion of pro-inflammatory cytokines, like Tumor Necrosis Factor-α (TNF-α), IL-1, and IL-6 (25). The above studies suggest that Gln may serve as a cofactor supporting normal macrophage function and may promote AKI progression by regulating macrophage phenotypic transformation.

In a study of obese mice with polymicrobial sepsis, intravenous administration of Gln after sepsis induction restored plasma Gln levels, reduced renal high mobility group box-1 (HMGB-1) pathway activation and macrophage/neutrophil infiltration, as indicated by CD68, EGF-like module-containing mucin-like hormone receptor-like 1 (EMR-1), and myeloperoxidase (MPO) expression, and increased tight junction protein zonula occludens-1 (ZO-1) levels (78). In animal models of this form of AKI, Gln supplementation may not only support tissue repair by providing metabolic substrates but also alleviate renal injury by modulating inflammatory signaling pathways. Therefore, targeting macrophage Gln metabolic pathways may reduce renal damage or delay renal fibrosis progression, which may represent a promising therapeutic strategy.

In summary, identifying immune metabolic pathways within immune cells through mechanistic studies may provide potential targets for preventing AKI or promoting renal recovery. For example, selectively targeting the metabolic dependencies of pathogenic effector cells may be a more valuable strategy than broad immunosuppression. In the future, strategies such as modulating Gln metabolism to suppress pathogenic effector T cells and M1 macrophages during the early phage, while promoting Treg and M2 phenotypes during the later stage, may enable cell- and stage-specific therapeutic approaches.

### 3.3 Gln and acid-base balance

The normal function of the kidneys is crucial for maintaining blood PH. Severe MA(pH < 7.2) occurs in over 5% of Intensive Care Unit (ICU) patients, with a mortality rate exceeding 50% (79, 80). MA and AKI often coexist and are associated with adverse clinical outcomes. Within this pathological interplay, Gln metabolism may serve as an important link connecting acid-base imbalance to renal damage (80).

Under conditions of MA, renal Gln uptake and metabolism undergo significant reprogramming. Approximately one-third of plasma Gln is taken up by the kidneys (55).In MA models, SNAT3 expression is upregulated and expands beyond its predominant localization in the S3 segment of the late PT to broader regions of the PT, thereby enhancing Gln utilization and ammonium production (55, 81, 82).

The absorbed Gln undergoes accelerated catabolism via GLS1 and GLUD. This process is further amplified under acidotic conditions: the acidic environment activates ζ-crystallin, which in turn binds to GLS1 mRNA, inhibits ribonuclease recruitment, and stabilizes the transcript, thereby enhancing its expression and metabolic flux. Consequently, Gln metabolism is accelerated, yielding increased amounts of α-KG, NH_4_^+^, HCO_3_^−^. The resulting ammonium ions are excreted in the urine, while bicarbonate is released into the bloodstream, collectively acting to alleviate systemic acidosis (83). α-KG is partially converted to 2-HG in hypoxic and acidic environments, which is involved in the accumulation and sustained activation of the HIF-α, which stimulates glycolysis metabolism and whose product, lactic acid, cannot replenish the TCA cycle. Thus, reversing the acidic environment also favors the restoration of the impaired anaplerosis (84).

MA is not only a common metabolic abnormality following AKI but may also predispose the kidney to AKI. Epidemiological data show that patients with CKD complicated by MA have a significantly elevated risk of developing AKI (85). The occurrence of this clinical phenomenon may involve multiple pathological mechanisms. Animal studies by Bugarski et al. (80) indicate that Gln metabolism may contribute to this process. Specifically, MA can induce AKI by disrupting NAD redox balance and lipid metabolism in the PT. During MA, the uptake and metabolism of Gln in the PT are markedly upregulated. This process is highly dependent on oxidized NAD^+^ as a cofactor, increasing NAD^+^ demand, reducing NAD(P)H availability, and shifting the NAD pool toward a more oxidized state. Consequently, normal fatty acid metabolism is suppressed. This metabolic disturbance triggered notable lipid accumulation and vacuolation in renal TECs, particularly within the S2 segment, in mouse models with diminished NAD(P)H signaling, thereby confirming the resultant lipid metabolic dysregulation and its associated tubular injury.

In summary, Gln metabolism represents an important adaptive pathway by which the kidney responds to MA, while metabolic reprogramming during AKI may further influence renal injury progression. Dysregulated Gln utilization may be associated with NAD metabolic imbalance and lipid metabolism disorders, thereby contributing to the progression of renal injury. These findings suggest that Gln metabolism may serve as a metabolic link between MA and AKI, however, its underlying mechanisms and clinical significance require further investigation.

### 3.4 The role of metabolic intermediates of Gln

At the onset of AKI, dysregulated Gln metabolism may lead to an imbalance of its downstream metabolites, which in turn affect redox balance, oxidative stress, and immune cell function and promote inflammatory responses. We hypothesize that this process not only exacerbates kidney injury but may also contribute to distant organ injury through inflammatory regulation and oxidative stress modulation. GSH, a downstream product of Gln, serves as a vital intracellular antioxidant by neutralizing ROS and preserving cellular redox homeostasis. At the onset of AKI, Gln depletion may reduce GSH synthesis, impair cellular antioxidant capacity, exacerbate oxidative stress, induce TEC necrosis and apoptosis, and promote ferroptosis (86). Zhan et al. used a rat model of gentamicin-induced AKI (87) and found that Gln supplementation increased the activities of catalase, superoxide dismutase and GSH peroxidase and attenuated oxidative stress and renal dysfunction. Mechanistically, GSH intervention reduces blood urea nitrogen (BUN) and malondialdehyde (MDA) levels and attenuated glomerular injury and renal structural damage. This is associated with downregulation of the pro-ferroptotic protein Acyl-CoA synthetase long chain family member 4 (ACSL4), inhibition of iron accumulation, and upregulation of the key ferroptosis inhibitor glutathione peroxidase (GPX4), thereby enhancing resistance to lipid peroxidation and inhibiting ferroptosis (86).

AKI not only induces renal injury but also triggers systemic metabolic reprogramming and may contribute to distant organ dysfunction. Using animal models, researchers found that AKI induces a complex interorgan metabolic communication network. Ischemic AKI triggers hepatic metabolic reprogramming, characterized by depletion of the Gln/Glu-α-KG axis and TCA cycle intermediates, accompanied by reduced GSH and its precursors, thereby impairing hepatic redox homeostasis (88). These metabolic alterations may represent a systemic metabolic adaptation after AKI rather than merely localized renal metabolic disturbances (88). 24 hours after ischemic AKI, the levels of Glu and GSH in mouse lung tissues were significantly reduced, indicating substantial consumption of these molecules during AKI-related pathological processes (89). Depletion of GSH may explain the biological mechanisms underlying the increased susceptibility and severity of pulmonary complications, such as acute respiratory distress syndrome (ARDS) (89). Similarly, experimental studies have shown that after AKI, the heart undergoes metabolic reprogramming similar to that observed in myocardial IRI, including disrupted amino acid metabolism, GSH depletion, and increased oxidative stress (90). However, evidence supporting the relevance of these findings to human disease remains limited.

Overall, current understanding of Gln metabolic reprogramming in AKI is largely based on experimental studies and focuses primarily on the early stages of injury. The mechanisms involved in later phases, including adaptive repair and maladaptive repair, remain to be further elucidated. These studies provide important insights into cellular responses and potential therapeutic mechanisms in AKI, however, further research is required to validate these findings in human AKI.

## 4 Gln metabolism in CKD

AKI carries a propensity to progress toward CKD, with maladaptive repair representing a core underlying mechanism. Recent research suggests that Gln metabolic reprogramming may constitute a significant metabolic hallmark of CKD and further serves as a key mechanism driving its central pathological process—renal fibrosis (RF). Compared with AKI, Gln metabolism alterations associated with CKD have been investigated in the clinical cohort, however, understanding of the underlying mechanisms remains largely based on preclinical studies.

At the level of circulating metabolites, significant metabolic changes were observed in the UUO animal model, manifested as abnormalities in lipid, amino acid and bile acid metabolism. Notably, plasma Glu levels were elevated (91). In 5/6 nephrectomized rats, serum Glu levels were found to be significantly elevated (92). In UUO rats with RF, serum L-Gln levels were significantly increased and showed a positive correlation with RF severity (93). Building on the preclinical evidence, this finding was further supported by analyses of clinical samples. To investigate the relationship with RF, Cai et al. collected serum and renal biopsy samples from patients diagnosed with IgA nephropathy exhibiting varying degrees of RF. Their analysis revealed that patients with severe fibrosis had significantly elevated serum Gln levels. Immunofluorescence further revealed enhanced GLS1 expression within the renal interstitium of these patients (20). Zhou et al. found that compared to healthy controls, patients with CKD exhibited an elevated plasma Glu/Gln ratio, suggesting increased GLS1 activity (94).

Data from animal models and human studies indicate that alterations in circulating Gln and its metabolites, the plasma Glu/Gln ratio, and tissue GLS1 expression suggest metabolic reprogramming of Gln metabolism in CKD. Although these reprogramming patterns may vary among CKD models, disease stages, and sample sources, Gln metabolism represents a critical component of the metabolic dysregulation in CKD. The alteration not only offers potential biomarkers for diagnosis and risk stratification but also provide a critical metabolic framework for elucidating the progression mechanisms of CKD.

### 4.1 Gln and renal fibrosis

Despite the diverse etiologies of CKD, RF represents a shared common pathway in nearly all cases, with its extent inversely correlating with renal function (95). Therefore, targeting fibrosis remains a key focus in CKD management; however, no established therapies are currently available to prevent or reverse fibrosis. Recent research has increasingly focused on the role of glutaminolysis in organ fibrosis. Across multiple organs, including the kidney, GLS1-mediated Gln metabolic reprogramming contributes significantly to the development of fibrosis (26). This reprogramming acts as an important metabolic nexus, linking chronic injury signals to the final fibrotic outcome, thereby positioning it as a potential common therapeutic target for intervention in fibrosis across various organs. Renal TECs typically act as early responders to fibrotic stimuli and important regulators of RF progression, while the activation of fibroblasts and myofibroblasts constitutes a key event (96). Using animal models and cell-based experiments, researchers have found that Gln may contribute to RF by participating in key processes such as fibroblast activation and macrophage modulation. **Figure 3** illustrates the role of Gln metabolism in RF.

**Figure 3:**
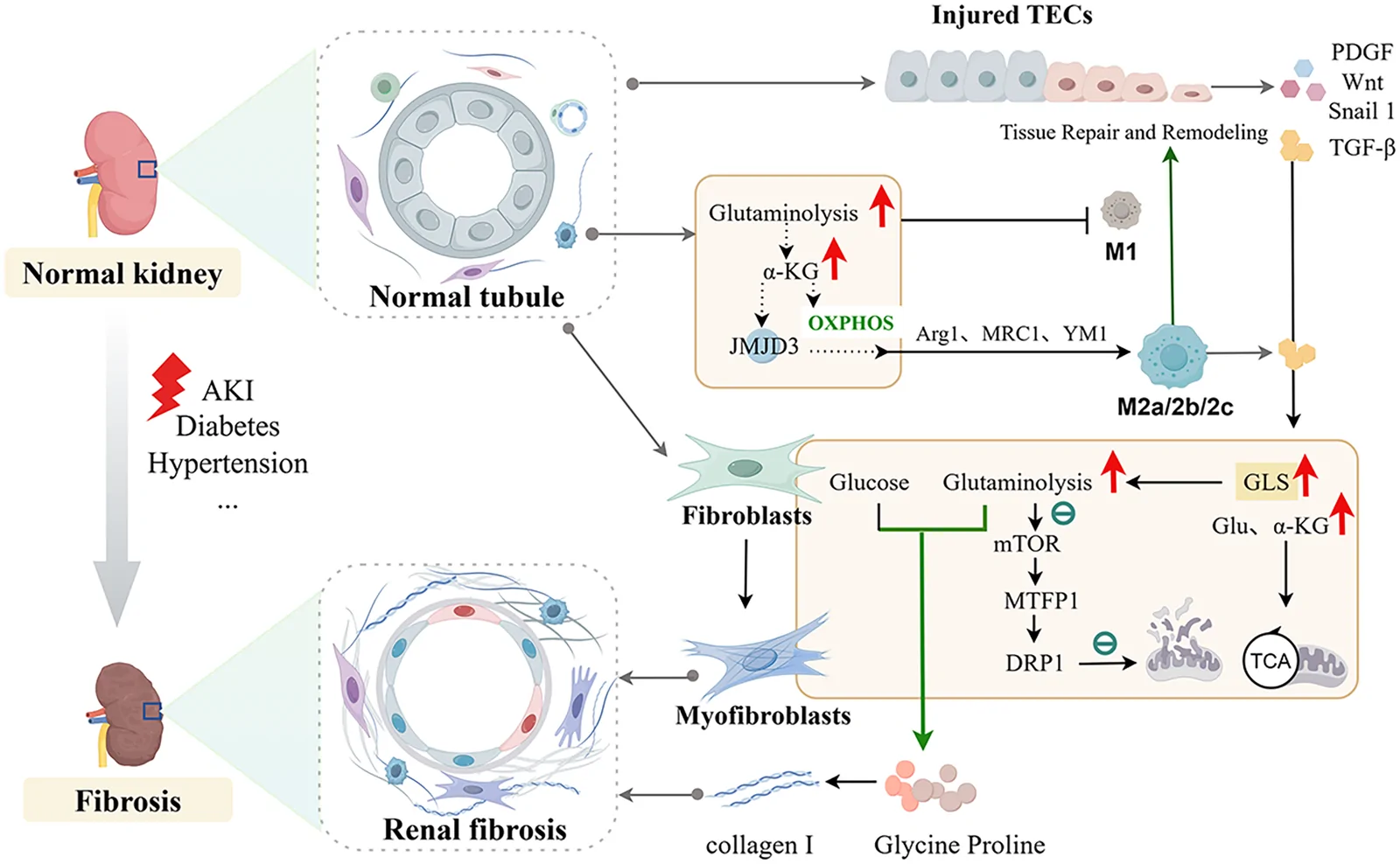
The mechanisms of Gln metabolism in driving the progression of renal fibrosis. Enhanced glutaminolysis in macrophages is closely associated with their polarization toward the reparative M2 phenotype. While moderate activation of M2 macrophages promotes tissue repair, their excessive activity contributes to renal fibrosis. The process of renal fibrosis is initiated by multiple factors, with fibroblasts serving as the central players. Compared to glucose metabolism, Gln metabolism plays a more critical role in fibroblast proliferation, migration, and secretion of extracellular matrix. Signaling molecules such as TGF-β, secreted by injured renal tubular epithelial cells and macrophages, activate the Gln metabolic pathway in fibroblasts. This activation generates various downstream metabolites. These metabolites not only participate in energy supply but also include key intermediates like α-ketoglutarate, which helps maintain mitochondrial function and morphology. Furthermore, α-ketoglutarate may regulate mitochondrial fission by modulating the mTOR/MTFP1/Drp1 pathway during fibroblast activation. AKI, acute kidney injury; Gln, Gln; GLS, Glutaminase; Glu, Glutamate; GDH, Glutamate Dehydrogenase; α-KG, α-Ketoglutarate; OXPHOS, Oxidative Phosphorylation; JMJD3, Jumonji Domain-Containing Protein 3; TECs, tubular epithelial cells; Arg1, Arginase 1; MRC1, Mannose Receptor C-Type 1; YM1, also known as chitinase-like protein 3, Chi3l3; mTOR, mammalian target of rapamycin; MTFP1, Mitochondrial Fission Process 1 Protein; DRP1, dynamin-related protein 1; PDGF, platelet-derived growth factor; Wnt, Wingless-related integration site; Snail1, encoding snail family zinc finger 1, known as Snail1; TGF-β, transforming growth factor- β. TCA, the tricarboxylic acid.

Cai et al. reported that *in vitro*, TGF-β1-induced activation of renal fibroblasts was accompanied by an upregulation of Gln metabolic pathways and an increase in key metabolites such as Glu and α-KG. Furthermore, Gln deprivation more potently suppressed fibroblast proliferation, migration, and extracellular matrix secretion compared to glucose deprivation, suggesting that fibroblasts maintaining a pro-fibrotic phenotype exhibit a greater dependence on Gln (20). This finding aligns with observations in liver fibrosis, where Gln deprivation or inhibition of GLS1 prevented the differentiation of quiescent hepatic stellate cells into myofibroblasts—an effect not observed with glucose deprivation (97). Mechanistically, Gln metabolism sustains mitochondrial function and morphology via its key intermediate α-KG, and may regulate mitochondrial fission by modulating the mechanistic target of rapamycin/mitochondrial fission regulator/dynamin-related protein 1(mTOR/MTFP1/Drp1) pathway during fibroblast activation. In a mouse model of RF induced by UUO, both pharmacological inhibition of GLS1 and conditional knockout of the gls gene in fibroblasts significantly attenuated renal interstitial fibrosis and the expression of fibrotic markers (20).

Macrophages also play a pivotal role in the inflammatory and fibrotic microenvironment of the kidney. During macrophage polarization toward the M1 phenotype, glycolysis is enhanced while oxidative phosphorylation (OXPHOS) is reduced, a metabolic shift associated with early inflammatory damage. In contrast, M2 macrophages maintain high levels of OXPHOS, which supports their tissue repair functions (98–100). If M2 macrophages remain overactive during the late stages of injury, they may contribute to RF and the progression of CKD (74, 101, 102). Therefore, the metabolic state of macrophages does not simply determine whether they are protective or pathogenic, rather, it influences disease progression by regulating their functional plasticity. In M2 macrophages, oxidative metabolism and Gln catabolism are major metabolic pathways supporting cellular functions (75). Gln is metabolized by GLS1 to generate α-KG, a key metabolic intermediate that links metabolic state with cellular function and plays an important role in M2 macrophage polarization (77). Therefore, Gln metabolism may indirectly influence fibrosis, and targeting macrophage Gln metabolism to modulate their functional plasticity may represent a potential strategy for managing inflammation and fibrosis in CKD. However, the therapeutic potential of this approach remains to be further explored. Studies in immunometabolism have shown that Gln metabolism regulates CD4^+^ T cell differentiation and effector functions by promoting metabolic reprogramming in activated T cells, including GLS1-dependent Gln catabolism, mTORC1/c-Myc signaling, and biosynthetic pathways (68, 103, 104). Its specific role in the immune microenvironment of CKD remains to be further elucidated. Current evidence also suggests that persistent intrarenal T-cell responses following AKI play an important role in renal repair and the transition from AKI to CKD (105–107). Enhanced Gln metabolism may sustain pathogenic Th17 responses in CKD by promoting GLS-dependent metabolic reprogramming, thereby exacerbating IL-17α-driven renal inflammation and fibrosis (103, 108, 109). Pharmacological inhibition of GLS1 reprograms T-cell metabolism and attenuates RF (72). Although direct evidence demonstrating that Gln metabolism regulates T-cell function during CKD progression remains limited, further investigation of this mechanism may provide new insights into slowing the AKI-to-CKD transition and delaying CKD progression. Immunometabolic regulation may represent a promising avenue for future research on the AKI-to-CKD transition.

### 4.2 Gln and cardiovascular complications

In addition to its local effects on the kidneys, CKD may also contribute to the development of CKD-related cardiovascular complications by inducing systemic reprogramming of Gln metabolism and thereby influencing the metabolic homeostasis of distant organs.

Cardiovascular disease represents one of the primary causes of mortality in patients with CKD, with arterial calcification being a highly prevalent pathological alteration (110, 111). Multiple studies indicate that dysregulated GLS1 is involved in metabolic remodeling in various models of age-related disorders and cardiovascular diseases (94). Emerging evidence suggests a potential role for GLS1 dysregulation in CKD-associated arterial calcification. Clinical sample analyses reveal that CKD patients exhibit significantly elevated plasma Glu levels and an increased Glu/Gln ratio, both of which correlate positively with the severity of aortic calcification, suggesting that Gln metabolism disorders may be associated with CKD-related vascular lesions (94). However, correlational studies alone cannot establish a direct causal relationship. In CKD mouse models, researchers have found that GLS1 expression is elevated in vascular calcification models, and that pharmacological inhibition of GLS1 attenuates osteogenic transdifferentiation of vascular smooth muscle cells (VSMCs) and reduces vascular calcification *in vivo*. Conversely, GLS1 overexpression or exogenous Glu supplementation promotes a calcific phenotype (94). Experimental findings provide mechanistic support for the involvement of GLS1 in CKD-associated vascular calcification. The role of Gln metabolism in vascular calcification in human CKD requires further investigation.

## 5 Gln metabolism as a potential therapeutic target in acute and chronic kidney diseases

### 5.1 Regulation of Gln availability in AKI

Currently, AKI prevention and treatment have shifted toward precise risk stratification, with an emphasis on early identification of high-risk individuals and multifaceted renal protection strategies, including balanced crystalloid use and appropriate fluid management (112–114). Treatment primarily focuses on supportive measures, including optimization of hemodynamic status, avoidance of nephrotoxic factors, fluid management, and renal replacement therapy (RRT) (112). As a vital organ for energy metabolism, the kidneys often exhibit significant metabolic dysfunction following injury. However, current management strategies lack specific interventions targeting metabolic abnormalities associated with AKI. Given the important role of metabolic reprogramming in tubular injury and immune regulation, interventions targeting metabolic abnormalities may represent a promising adjunctive therapeutic approach. A large multicenter randomized clinical trial (RCT) published in 2024 demonstrated that perioperative intravenous amino acid administration during cardiac surgery significantly reduced the incidence of postoperative AKI (115). This high-quality evidence not only underscores the importance of perioperative metabolic optimization but also highlights amino acid metabolism as a promising therapeutic target. Collectively, this evidence suggest that metabolic modulation may serve as a valuable adjunct to current AKI management strategies.

Previous studies have suggested that exogenous Gln supplementation may serve as a potential metabolic support strategy with potential clinical benefits (116). Gln serves as an important metabolic substrate involved in tissue energy metabolism and stress responses. Under acute stress, reduced Gln availability may impair metabolic adaptation following organ injury. Existing studies have investigated the therapeutic potential of Gln supplementation in various acute disease models and clinical settings, with relevant clinical studies summarized in **Table 1**. However, the efficacy of Gln supplementation for AKI prevention and treatment requires further validation through both experimental and clinical evidence.

**Table 1:** Clinical application of glutamine supplementation for different acute diseases.

Disease	Study design	Glutamine dosage	Mode of administration	Key findings/effects	References
Acute kidney injury after cardiac surgery	Randomized controlled trial	0.5 g/kg over 12 hours	Intravenous Glutamine	Reduced markers of kidney damage	(19)
Severe burns	Double-blind Randomized controlled trial	0.5 g/kg/day	Enteral Glutamine (feeding tube or oral administration)	No significant effect	(117)
Severe Acute pancreatitis	Systematic review and Meta-Analysis	Various doses among included RCTs	Enteral or parenteral Glutamine	Improved prognosis and reduced infectious complications and mortality	(118, 119)
Sickle cell disease	Randomized controlled trial	0.3 g/kg/dose every 12 h	Oral Glutamine	Reduced frequency and severity of vaso-occlusive crises	(120)
Acute myocardial infarction with heart failure	Randomized controlled trial	10 g, three times a day	Intravenous Glutamine	Improved intestinal barrier function and cardiac function	(121)

Current clinical studies on the application of glutamine supplementation in acute diseases and acute complications. Dosages and administration routes vary depending on the disease and study protocol. IBF, intestinal barrier function.

#### 5.1.1 Potential role of Gln supplementation in CSA-AKI prevention

Gln supplementation has recently attracted attention as a potential metabolic strategy for preventing AKI associated with cardiac surgery. During cardiopulmonary bypass (CPB), systemic inflammation, oxidative stress, IRI injury, and metabolic abnormalities may all contribute to the development of AKI (122). As an important metabolic substrate under stress, changes in Gln homeostasis may play a role in the process of perioperative kidney injury. A small RCT found that Gln levels were significantly lower after CPB than before surgery (19). In high-risk cardiac surgery patients, Gln administration not only provided cardioprotective effects but also reduced renal stress and injury biomarkers. Although this small-scale trial found no significant difference in postoperative AKI incidence, larger clinical studies are needed to determine whether Gln can reduce cardiac surgery-associated AKI (CSA-AKI) or improve long-term renal outcomes. Nevertheless, this finding suggest that Gln may have the potential to prevent perioperative AKI in high-risk cardiac surgery patients. A pooled analysis of two RCTs found that perioperative Glu supplementation in patients undergoing coronary artery bypass grafting (CABG) was associated with a lower incidence of postoperative AKI, although it did not affect cardiac injury markers (123). These findings suggest that Gln-based metabolic support during the perioperative period of cardiac surgery may represent a potential strategy for preventing postoperative complications. Given that Gln/Glu levels may reflect metabolic abnormalities and immune status in patients with cardiovascular disease (124), future clinical studies should investigate whether alterations in Gln metabolism and this ratio can serve as biomarkers for predicting postoperative AKI risk and identifying patients most likely to benefit from metabolic interventions.

Given the limited evidence currently, caution is warranted when extrapolating findings from cardiac surgery to other surgical fields. For example, the mechanisms underlying AKI may differ across procedures, different disease states may be associated with distinct Gln metabolic profiles, and Gln requirements may also vary. Therefore, the abnormal Gln metabolism observed after cardiac surgery and the preventive effects of exogenous supplementation cannot yet be directly extrapolated to other surgical procedures. However, existing studies suggest that perioperative Gln supplementation may modulate postoperative recovery by regulating metabolic homeostasis. Its potential clinical utility in preventing surgery-related AKI warrants further investigation. Future research should identify patient subgroups likely to benefit and optimize intervention doses, routes of administration, and treatment timing, thereby generating translational evidence to support metabolism-based precision intervention strategies in surgical patients.

#### 5.1.2 Potential role of Gln supplementation in AKI treatment

Recent research efforts have focused on exploring the therapeutic potential and molecular mechanisms of Gln in the context of AKI. **Table 2** summarizes a series of experimental studies that used various animal models to investigate the beneficial effects of exogenous Gln supplementation on renal function in the early stages of AKI. In a gentamicin-induced AKI rat model, Gln demonstrated renal protective effects through its anti-inflammatory and antioxidant properties. These effects were evidenced by reduced ROS levels and increased levels of catalase, superoxide dismutase (SOD), GPX, and GSH (87). In an obese mouse model of polymicrobial sepsis (CLP), where inflammation is the primary driver, Gln administration alleviated inflammatory responses and reduced markers of kidney injury (78). Gln supplementation can mitigate oxidative stress and apoptosis, thereby improving renal function and alleviating tissue damage following AKI. This is achieved by upregulating Hsp70 expression, inhibiting Ask1 activation, and reducing mitochondrial apoptotic pathway activation in renal TECs (14). Furthermore, Gln mitigates cisplatin-induced renal injury through multiple mechanisms, such as reducing cisplatin accumulation by modulating organic cation transporter 2 (OCT2) expression and regulating oxidative stress by stabilizing GSH levels (59). These studies provide a mechanistic basis for Gln as a metabolic intervention strategy for AKI. However, the evidence supporting the therapeutic benefits of Gln supplementation is primarily preclinical, and its efficacy in patients with AKI remains uncertain.

**Table 2:** Summary of studies on the application of glutamine and its metabolites in AKI.

Treatment	Study type	AKI model	Dosage	Mode of administration	Treatment time	Renal outcomes	Key findings	Reference
Glutamine supplement	Experimental study	Polymicrobial sepsis-induced kidney injury in mice	41.7g/kg experimental diets	Glutamine-enriched diet	2 weeks before CLP induction and continued until 72 h after CLP	Kim-1, MIP-2, KC expression↓, Bcl-2 expression↑	Balanced T cell polarization and attenuated kidney injury	(73)
	Experimental study	APAP-induced rat model of AKI	0.75 mg/kg	Intraperitoneal injection	Single dose administered 30 min before APAP	GFR↑, BUN↓, SCr↓	Preserved basolateral localization of Na^+^, K^+^-ATPase and attenuated renal structural injury	(125)
	Experimental study	Gentamicin-induced rat model of AKI	50 mg/kg and 500 mg/kg	Oral Glutamine	14 consecutive days after AKI induction	RBF↑, CCr↑, BUN↓, serum creatinine↓	Reduced oxidative stress and enhanced antioxidant defense	(87)
	Experimental study	Renal IRI-induced AKI mouse model	0.5 g/kg	Intravenous Glutamine	Single dose 4 h after reperfusion	NGAL↓, KIM-1↓, [TIMP-2]*[IGFBP7]↓, BUN↓, SCr↓, alleviated apoptosis	Oxidative phosphorylation↑, NAD+/NADH ratio↑, JNK activation, improved mitochondrial function↓	(14)
	Experimental study	Polymicrobial sepsis-induced AKI in obese mice (CLP model)	0.75 g/kg	Intravenous Glutamine	1 h after CLP; additional dose at 24 h in 48-h group	NGAL↓, Kim-1 (48 h) ↓; no significant change in BUN/creatinine	Reduced renal macrophage and neutrophil infiltration, attenuated inflammatory signaling	(78)
	Randomized controlled trial	CSA-AKI (high-risk patients)	0.5 g/kg over 12 hours	Intravenous Glutamine	Started after CPB termination, after biomarker	urinary [TIMP-2]*[IGFBP7]↓, KIM – 1↓, NGAL↓	Reduced kidney stress and injury biomarkers; no significant reduction in AKI incidence	(19)
Glutamate supplement	Pooled analysis of 2 RCTs	CSA-AKI after CABG	0.125-M L-glutamic acid (1.65 mL/kg/h)	Intravenous Glutamate	During reperfusion after aortic cross-clamp release	Risk of SCr increase ≥50% ↓, dialysis rate ↓	Risk of AKI decreases	(123)
Glutathione supplement	Experimental study	Renal IRI-induced AKI mouse model	800 mg/kg	Intraperitoneal injection	1 h after successful AKI modeling, repeated daily for 6 consecutive days	BUN↓, MDA↓, lipid oxide levels↓	ACSL4 expression↓GPX4 expression↑, reduced iron accumulation, inhibited ferroptosis and alleviated renal injury	(86)

AKI, acute kidney injury; KIM-1, kidney injury molecule-1; MIP-2, macrophage inflammatory protein-2; KC, keratinocyte chemoattractant; Bcl-2, B-cell lymphoma 2; APAP, acetaminophen; GFR, glomerular filtration rate; BUN, blood urea nitrogen; SCr, serum creatinine; RBF, renal blood flow; CCr, creatinine clearance; Cr, creatinine; SOD, superoxide dismutase; GPX, glutathione peroxidase; IRI, ischemia-reperfusion injury; NGAL, neutrophil gelatinase-associated lipocalin; [TIMP-2]·[IGFBP7], product of tissue inhibitor of metalloproteinases-2 and insulin-like growth factor-binding protein 7; JNK, c-Jun N-terminal kinase; CSA-AKI, cardiac surgery-associated acute kidney injury; CPB, cardiopulmonary bypass; CABG, coronary artery bypass grafting; MDA, malondialdehyde; ACSL4, acyl-CoA synthetase long-chain family member 4.

In critical care, clinical studies on Gln supplementation have yielded inconsistent results (126–128). Specifically, the early, separate administration of high-dose Gln failed to demonstrate benefit for critically ill patients with multiple organ failure and was even associated with increased mortality (129). This discrepancy in outcomes may be attributed to the indiscriminate administration of supplemental Gln to all patients without consideration of their baseline plasma levels. Plasma Gln concentration serves as a prognostic biomarker in critically ill patients upon ICU admission. Deviations from the normal range, whether higher or lower, have been associated with increased mortality (127). In one study that employed an intravenous low-dose isotopic tracer technique to measure Gln flux, low plasma Gln levels at ICU admission were linked to insufficient endogenous production. This finding suggests that targeted supplementation for this specific patient subgroup may be necessary to maximize therapeutic benefit (130).

For AKI, the association between plasma Gln concentration and clinical outcomes in critically ill patients with AKI remains unclear. Given the potential risks of Gln supplementation in critically ill patients, this association must be clarified before the mechanism can be translated into clinical management strategies for AKI. Not all patients with AKI will benefit from indiscriminate Gln supplementation, those with low plasma Gln levels and insufficient endogenous Gln production may benefit the most. The emergence of point-of-care Gln monitors may facilitate clinical implementation of this approach (131). It remains unclear whether a threshold exists in patients with AKI such that appropriate, rather than excessive, Gln supplementation helps them withstand acute stress, increases energy reserves, and enables survival of the acute injury phase. Therefore, future research should first stratify patients with AKI by levels of Gln and its metabolites and examine their associations with outcomes across different etiologies. Such observational studies will provide a foundation for identifying the optimal patient population and therapeutic window for Gln supplementation. Well-designed clinical trials can then validate these findings. AKI involves multiple sequential pathological stages, and it remains unclear at which stage exogenous Gln supplementation would provide the greatest benefit. In summary, the therapeutic efficacy of Gln supplementation may be influenced by multiple factors, including baseline patient characteristics, disease severity, timing of administration, dosage, and route of delivery. Currently, the protective effects observed in experimental AKI models cannot be directly extrapolated to all AKI patients, however, further investigation is warranted. Combining metabolic phenotyping with biomarker-based stratification may improve the efficiency and design of future clinical trials. Gln supplementation strategies should not be guided solely by disease diagnosis, rather, they should be tailored to the patient’s metabolic phenotype and disease stage.

### 5.2 Targeting Gln metabolism in CKD

Increasing experimental evidence indicates that Gln metabolic reprogramming plays a key metabolic role in regulating fibroblast differentiation, therefore, GLS1 is considered a potential therapeutic target for multiorgan fibrosis (132). Inhibition of GLS1 exerts antifibrotic effects by impairing the metabolic adaptability of activated fibroblasts (133). Moreover, GLS1 activity is functionally linked to key pro-fibrotic signaling pathways, including TGF-β and nuclear factor kappa-light-chain-enhancer of activated B cells (NF-κB) (134). GLS1 inhibition elicits anti-fibrotic effects through the mechanistic pathways described above. For instance, In a mouse model of bile duct ligation, Ganfule capsules attenuate liver fibrosis, potentially through inhibition of the NF-κB signaling pathway and downregulation of GLS1 expression (134). In pulmonary fibrosis models, the GLS1 inhibitor telaglenastat (CB-839) selectively suppress collagen synthesis while promoting its degradation (135). In cardiac fibrosis models, treatment with a GLS1 inhibitor reduces fibrosis area, collagen deposition, cardiomyocyte hypertrophy, and cardiac inflammation through the β-AR/Gls1/α-KG/KCa3.1 axis (136). Taken together, experimental findings from liver, lung, and heart fibrosis models provide a rationale for further investigating the role of GLS1 inhibition in RF.

Targeting GLS1 may represent a promising therapeutic strategy for the prevention and attenuation of RF. Studies by Cai et al. demonstrated that pharmacological inhibition of GLS1 in UUO mice significantly alleviated renal interstitial fibrosis and reduced the expression of fibrotic markers. Similarly, fibroblast-specific GLS1 knockout mice exhibited markedly attenuated RF and decreased extracellular matrix deposition. *In vitro* experiments further confirmed that inhibiting GLS1 significantly suppressed fibroblast activation (20). RF is an important driver of AKI-to-CKD progression, however, current antifibrotic strategies, such as TGF-β pathway inhibition, have shown limited efficacy (95). Experience with the development of antifibrotic therapies for pulmonary fibrosis has shown that targeting a single signaling pathway is often insufficient to effectively control the complex fibrotic process. Against this backdrop, targeting key enzymes such as GLS1 provides a novel metabolic perspective and may represent a complementary strategy to existing renoprotective or antifibrotic therapies. Johmura et al. used RNAi screening to demonstrate that the Gln degradation pathway, particularly its rate-limiting enzyme GLS1, is essential for the survival of senescent cells. Clearance of senescent cells or inhibition of Gln degradation may alleviate age-related organ dysfunction (26, 137). Because cellular senescence accelerates the progression of kidney diseases such as CKD (138), Gln degradation may also represent a potential therapeutic target for CKD. However, its therapeutic potential and clinical applicability require further validation.

Additionally, Glu dehydrogenase 1 (GDH1), an enzyme within the same Gln metabolic pathway, is downregulated in fibrotic kidneys. Despite this decrease, inhibiting its residual activity alleviates fibrosis by relieving suppression of peroxisome proliferator-activated receptor gamma (PPARγ), suggesting GDH1 as another potential therapeutic target in RF, with 2-allyl-1-hydroxy-anthraquinone (R162) representing a promising lead compound (139). Notably, studies in pulmonary fibrosis indicate that targeting GLS1 alone may be insufficient due to metabolic compensation by GDH1, highlighting that effective intervention in this metabolic pathway likely requires a more integrated approach (133).

### 5.3 Targeting Gln-mediated immunometabolic regulation in acute and chronic kidney diseases

Unlike exogenous supplementation for AKI or strategies targeting Gln metabolism in RF, immunometabolic regulation has emerged as a novel strategy for modulating the renal injury microenvironment (140). Since immune cells are highly dependent on Gln metabolism to maintain activation, proliferation, and effector functions, targeting Gln metabolism in immune cells may offer a novel therapeutic approach for regulating inflammatory responses and restoring dysregulated tissue repair. As illustrated in **Figure 4**, Gln-mediated immunometabolic remodeling represents a potential regulatory axis linking immune responses and tissue repair across different stages of kidney disease. Understanding the context-dependent functions of this pathway may provide a rationale for developing stage- and cell-specific therapeutic strategies targeting Gln metabolism. Experimental evidence suggests that the GLS1 inhibitor JHU083 reduces T-cell activation in IRI- and cisplatin-induced AKI models by limiting Gln utilization by T cells, thereby ameliorating renal injury markers (71). During the recovery phase following severe AKI, inhibiting Gln metabolism to attenuate T-cell metabolic reprogramming effectively reduces RF and effector CD4+ T-cell activation (72). Therefore, this strategy may slow AKI-to-CKD progression and mitigate other contributors to CKD development. Macrophages can regulate fibrosis by modulating Gln metabolism, suggesting that targeting this pathway may help attenuate RF progression (141). The underlying mechanisms may involve immune cell functional remodeling or altered crosstalk between immune cells and fibroblasts or mesenchymal cells, which requires further investigation (141). In a UUO mouse model, Gln antagonists such as 6-Diazo-5-oxo-L-norleucine (DON) attenuated RF and broadly reshaped the renal immune microenvironment (142). In DON-treated kidneys, collagen and CD52 levels were reduced, whereas macrophage migration inhibitory factor expression was increased. Furthermore, this approach induced kidney-infiltrating T cells to adopt a regulatory Treg phenotype, increased the proportion of senescent neutrophils with apoptotic features, alleviated UUO-induced hypoxia, and enhanced oxidative phosphorylation in TECs (142). These studies suggest that targeting Gln metabolism in immune cells may offer a novel approach for regulating the renal immune microenvironment and maladaptive repair processes. However, this strategy still faces several challenges, including how to distinguish pathological immune activation from reparative immune responses, minimize the off-target effects of systemic GLS1 inhibition, and determine the optimal intervention window across different disease stages.

**Figure 4:**
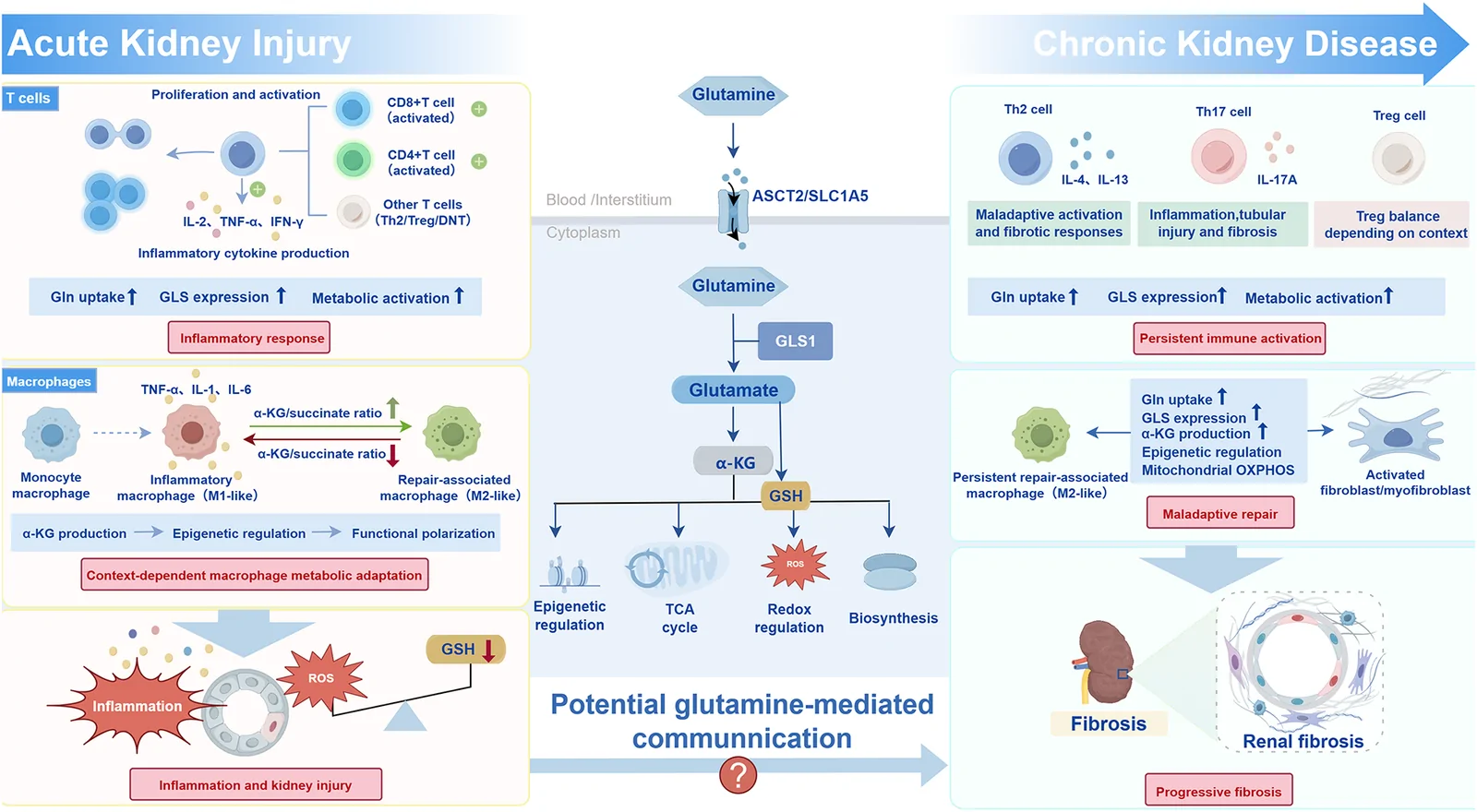
Context-dependent roles of Gln metabolism in immune modulation in AKI and CKD progression. This schematic illustrates the potential context-dependent roles of glutamine-mediated immunometabolic adaptation in AKI and CKD. During AKI, enhanced glutamine utilization through ASCT2/SLC1A5 and GLS1 supports T-cell activation and inflammatory responses, while macrophages undergo metabolic adaptation that may contribute to inflammation and kidney injury. During CKD, persistent glutamine-dependent immune programs may sustain chronic immune activation and persistent repair-associated macrophage (M2-like) activation, potentially contributing to maladaptive repair and progressive renal fibrosis. The contribution of glutamine metabolism to immune regulation and renal disease progression across the AKI-to-CKD continuum remains incompletely understood. Therapeutic targeting of glutamine metabolism may therefore exert stage- and context-dependent effects during AKI-to-CKD progression. Th2, T helper 2 cell; Treg, regulatory T cell; DNT, double-negative T cell; IL-2, interleukin-2; TNF-α, tumor necrosis factor-α; IFN-γ, interferon-γ; α-KG, α-ketoglutarate; ROS, reactive oxygen species; ASCT2/SLC1A5, alanine-serine-cysteine transporter 2/solute carrier family 1 member 5; GLS1, glutaminase 1; GSH, glutathione; TCA cycle, tricarboxylic acid cycle; IL-4, interleukin-4; IL-13, interleukin-13; IL-17A, interleukin-17A; Gln, glutamine; OXPHOS, oxidative phosphorylation.

The mechanisms underlying the AKI-to-CKD transformation remain an active area of research. Gln serves as both a metabolic substrate and an immunoregulatory molecule, the distinct metabolic alterations observed in AKI and CKD suggest that Gln metabolism may undergo dynamic changes during disease progression that remain to be characterized. However, as discussed above, current mechanistic studies suggest that these changes may be cell type-specific and stage-specific. Differences may exist in Gln metabolic dependence and responses among different immune cell types, as well as between immune cells and renal parenchymal cells. Most studies are limited to *in vitro* models and lack *in vivo* evidence at single-cell resolution. These differences, as well as the dynamic patterns of immunometabolic interactions across disease stages, remain unclear, current research has not yet fully elucidated the causal mechanisms underlying the transition from failed repair to fibrosis. Therefore, the dynamic changes in the Gln metabolic axis during the AKI-to-CKD transformation and the associated regulatory pathways warrant further investigation. The application of emerging technologies, such as single-cell sequencing and spatial metabolomics, is expected to reveal cell-type-specific metabolic reprogramming events. These approaches may also help identify metabolic pathways with therapeutic potential throughout disease progression. In addition, some studies are using approaches such as conditional gene knockout and metabolic flux tracing to strengthen causal evidence, moving beyond observational findings and associations toward mechanistic explanations.

In summary, interventions targeting Gln metabolism may serve as potential adjunctive metabolic therapies for AKI and CKD. However, the mechanisms underlying changes in Gln metabolism during AKI-to-CKD progression remain unclear. In addition to supportive care, perioperative renal protection, antifibrotic therapies, and immune cell-based interventions, additional therapeutic strategies warrant further exploration. Before clinical translation, it is necessary to thoroughly elucidate the underlying mechanisms, clarify metabolic compensation mechanisms, and systematically evaluate patient selection, treatment timing, dosing, and long-term safety. Therefore, Gln metabolism-based therapy is more likely to function as an adjunctive intervention alongside standard treatment rather than as an independent strategy, and its clinical potential requires further validation through combination approaches.

## 6 Limitations and future directions

The etiology of AKI and CKD is highly complex, exploring the potential common mechanisms or pathways is crucial for the further understanding of the pathogenesis. Gln metabolic reprogramming has provided a novel perspective in this field. Despite increasing evidence supporting the role of Gln metabolism in kidney disease, several limitations remain in our current understanding of this field. An in-depth analysis of stage-specific changes in Gln metabolism during kidney disease will not only facilitate the identification of dynamic biomarkers reflecting different pathophysiological states but also emphasize the importance of stage-specific treatment strategies. Future research should focus on the following issues.

### 6.1 Limitations of extrapolating mechanistic findings from heterogeneous models

Currently, most evidence regarding Gln metabolic reprogramming mechanisms in kidney injury is derived from experimental models. Since these models represent distinct pathological processes and metabolic environments, differences may exist in Gln metabolic patterns and responses to Gln-targeted interventions. Consequently, findings derived from a single experimental model cannot be directly extrapolated to AKI and CKD, which are characterized by substantial clinical heterogeneity. Future research should integrate multiple complementary disease models with well-characterized clinical cohorts to identify shared mechanisms across diseases with different etiologies, thereby improving the translational relevance of these findings.

### 6.2 Discrepancies between circulating metabolites and local renal metabolism

Current studies primarily assess metabolic alterations associated with AKI and CKD by analyzing changes in blood or urinary metabolites. These markers are clinically accessible and can reflect systemic metabolic alterations as well as disease risk. However, the relationship between these markers and local renal metabolic reprogramming, particularly across distinct renal cell types, remains poorly understood. Future research in this field should integrate circulating metabolomics, renal tissue metabolomics, spatial metabolomics, and single-cell analysis to characterize the relationship between systemic metabolic alterations and local renal metabolism.

### 6.3 Limited understanding of dynamic metabolic information

Current studies on renal Gln metabolism are largely based on single time-point measurements and lack metabolic flux analyses. This limitation makes it difficult to capture metabolic dynamics during AKI onset, recovery, and AKI-to-CKD progression, as well as to determine which cell types primarily utilize Gln and how Gln is distributed across different metabolic pathways. In the future, combining longitudinal metabolomics with serial assessment of Gln, Glu, GSH, α-KG, and related metabolites may help define more precise metabolic phenotypes and identify critical metabolic windows across disease stages. Additionally, integrating metabolic flux analysis approaches, such as stable isotope tracing, will help elucidate Gln utilization pathways in different cell types. Single-cell transcriptomic and spatial omics analyses will contribute to a comprehensive understanding of this process at cellular and molecular levels.

### 6.4 Cell-Type specificity of Gln metabolism

Furthermore, analysis of overall metabolite levels alone has limitations, as these changes may reflect the combined metabolic responses of multiple cell populations across different disease stages without determining the contribution of specific cell types. Gln metabolic regulation may vary substantially among different kidney cell types. Future research should integrate single-cell transcriptomics, spatial metabolomics, and cell-specific genetic models to elucidate cell-specific differences in Gln metabolic regulatory networks among TECs, fibroblasts, macrophages, and T cells, thereby defining their contributions to AKI and CKD progression. Furthermore, given the high metabolic plasticity of renal cells, targeting Gln metabolism may activate alternative metabolic pathways as a compensatory response, therefore, future studies should characterize metabolic network adaptations.

### 6.5 Limited clinical evidence and heterogeneity across etiologies

Clinical evidence supporting Gln metabolism-targeted interventions remains relatively limited. Existing clinical studies have primarily focused on perioperative Gln supplementation in cardiac surgery patients. Patients with AKI from different etiologies exhibit distinct injury mechanisms and metabolic characteristics, with substantial differences potentially existing in Gln metabolic profiles and related metabolite alterations. However, systematic research across different AKI subtypes remains limited, and it is unclear which patients are most likely to benefit from Gln-targeted interventions. Therefore, future studies should incorporate comprehensive stratification based on disease etiology, stage, and levels of Gln and related metabolites, while exploring associations between these factors and clinical outcomes to identify optimal patient populations and therapeutic windows. Other potential therapeutic strategies targeting the Gln metabolic pathway remain at the preclinical stage. In CKD, targeting key enzymes in the Gln metabolic pathway, including GLS1 and GDH, or modulating metabolism in specific immune cell populations may help slow RF progression. Additionally, GLS1 involvement in cellular senescence suggests that GLS1 inhibition may modulate CKD progression through aging-related pathways. This mechanism remains largely unexplored and warrants further investigation.

### 6.6 Limitations in biomarker translation

The clinical use of amino acid metabolism-derived biomarkers is hampered by variations in sample processing, analytical methods, and standardization procedures, which may lead to inconsistent findings and limit their translational application. In the future, these limitations may be addressed through standardized protocols for biospecimen collection and analysis, interlaboratory calibration, standardized reporting methods, and validation of biomarkers in populations with detailed dietary and comorbidity data. This would facilitate the clinical application of amino acid-based biomarkers. Other studies have also shown that multi-biomarker approaches hold promise for improving the accuracy of predicting disease onset and progression.

### 6.7 Potential role of Gln in regulating acid-base homeostasis

The roles of continuous renal replacement therapy (CRRT) and bicarbonate in the management of AKI-associated acidosis remain unclear. Given the potential role of Gln in regulating acid-base homeostasis, Gln-based interventions may represent a novel therapeutic avenue for AKI.

## 7 Conclusion

In summary, building on our analysis of changes in Gln metabolism during AKI and CKD, we have further examined its roles in TEC protection, inflammation regulation, immune cell function modulation, and tissue repair processes. Current research suggests that Gln metabolism does not merely reflect metabolic adaptation during kidney injury but may serve as a key regulatory node linking metabolic status, immune activation, and fibrotic progression. By analyzing multiple dimensions, including disease stage, cell type, and immunometabolic interactions, this paper offers a framework for future studies focusing on metabolic phenotypes, biomarkers, and precision therapeutic strategies.
